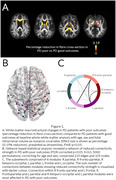# Multimodal neuroimaging and plasma biomarker evidence of white matter macrostructure loss in Parkinson's dementia

**DOI:** 10.1002/alz.093787

**Published:** 2025-01-09

**Authors:** Angeliki Zarkali, Naomi Hannaway, Peter McColgan, Amanda J Heslegrave, Elena Veleva, Rhiannon Laban, Henrik Zetterberg, Andrew Lees, Nick C Fox, Rimona S Weil

**Affiliations:** ^1^ Dementia Research Centre, UCL Queen Square Institute of Neurology, London United Kingdom; ^2^ Dementia Research Centre, University College London, London United Kingdom; ^3^ Huntington's Disease Centre, University College London, London United Kingdom; ^4^ UK Dementia Research Institute at UCL, London United Kingdom; ^5^ Institute of Neurology, University College London, London United Kingdom

## Abstract

**Background:**

Parkinson’s (PD) is common and debilitating with over half of patients progressing to postural instability, dementia or death within 10 years. However, onset and rate of progression is highly variable, reflecting heterogeneity in underlying pathology, and biomarker studies to‐date have been limited to a single modality or assessed patients with established cognitive impairment.

**Method:**

We assessed multimodal neuroimaging and plasma biomarkers in 98 PD patients (mean disease duration at baseline 4.33 years) and 28 age‐matched controls followed‐up over 3 years. Of PD patients, 31 (31.6%) had a poor outcome (PD poor outcomes), defined as mild cognitive impairment, dementia, frailty, or death during follow‐up. We compared biomarkers of gray matter (cortical thickness), white matter macrostructure (fibre cross‐section) and microstructure (fibre density) at whole‐brain and tract level, structural and functional connectivity, and plasma levels of neurofilament light chain (NFL) and phosphorylated tau 181 (p‐tau) between PD who progress to poor outcomes versus those with good outcomes.

**Result:**

We found extensive white matter macrostructural changes in PD who progress to poor outcomes already at baseline: with 19% reduction in fibre cross‐section and a subnetwork of reduced structural connectivity (105 nodes, 215 edges, p=0.017), particularly involving interhemispheric connections. Gray matter and functional connectivity were preserved. NFL but not p‐tau was increased in PD poor outcomes and correlated with white matter loss.

**Conclusion:**

PD patients who progress to poor outcomes, show white matter macrostructural loss already at baseline, evident both from imaging and fluid biomarkers. Our findings highlight white matter imaging and plasma NFL as potential biomarkers of poor outcomes in Parkinson’s, informing the design of future clinical trials.